# Dose-volume metric-based prediction of radiotherapy-induced lymphocyte loss in patients with non-small-cell lung cancer treated with modern radiotherapy techniques

**DOI:** 10.1016/j.phro.2024.100593

**Published:** 2024-05-27

**Authors:** Zuzanna Nowicka, Kasper Kuna, Mateusz Łaszczych, Małgorzata Łazar-Poniatowska, Bartosz Kamil Sobocki, Konrad Stawiski, Michał Dąbrowski, Konrad Bruski, Adam Zięba, Mateusz Pajdziński, Emilia Staniewska, Marcin Miszczyk, Harald Paganetti, Wojciech Fendler, Bartłomiej Tomasik

**Affiliations:** a Department of Biostatistics and Translational Medicine, Medical University of Łódź, Łódź, Poland; b Department of Oncology and Radiotherapy, Medical University of Gdańsk, Faculty of Medicine, Gdańsk, Poland; c Department of Radiotherapy, Medical University of Łódź, Łódź, Poland; d 3^rd^ Radiotherapy and Chemotherapy Department, Maria Skłodowska-Curie National Research Institute of Oncology, Gliwice, Poland; e Collegium Medicum, Faculty of Medicine, WSB University, Dąbrowa Górnicza, Poland; f Massachusetts General Hospital, Boston, MA, USA; g Harvard Medical School, Boston, MA, USA; h Department of Radiation Oncology, Dana-Farber Cancer Institute, Boston, MA, USA

**Keywords:** Radiotherapy, Lymphopenia, Non-small cell lung cancer

## Abstract

**Background and Purpose:**

Radiation-induced lymphopenia (RIL) is a common side effect of radiotherapy (RT) that may negatively impact survival. We aimed to identify RIL predictors in patients with non-small-cell lung cancer (NSCLC) treated intensity modulated radiotherapy (IMRT) and volumetric modulated arc therapy (VMAT).

**Materials and Methods:**

We retrospectively analysed data of 306 patients who underwent radical RT for NSCLC. Absolute lymphocyte count (ALC) loss was evaluated for each patient by fitting an exponential decay curve to data from first 45 days since treatment start, and percentage ALC loss relative to baseline was calculated based on area under the decay curve and baseline ALC. We compared IMRT and VMAT treatment plans and used linear regression to predict ALC loss.

**Results:**

ALC decreased during RT in the whole patient group, while neutrophil counts remained stable and decreased only in those treated with concurrent chemoradiotherapy (CRT). Percentage ALC loss ranged between 11 and 78 % and was more strongly than lymphocyte nadir correlated with dose-volume metrics for relevant normal structures. We found evidence for the association of high radiation dose to the lungs, heart and body with percentage ALC loss, with lung volume exposed to 20–30 Gy being most important predictors in patients treated with IMRT. A multivariable model based on CRT use, baseline ALC and first principal component (PC1) of the dose-volume predictors showed good predictive performance (bias-corrected R^2^ of 0.40).

**Conclusion:**

Percentage lymphocyte loss is a robust measure of RIL that is predicted by baseline ALC, CRT use and dose-volume parameters to the lungs, heart and body.

## 1 Introduction

Every year, approximately 2 million patients worldwide are diagnosed with lung cancer [1]. An estimated 75 % of these patients have an evidence-based indications for curative or palliative radiotherapy (RT) [2]. Despite the development of modern, conformal RT techniques normal tissues and circulating immune cells still receive non-negligible radiation doses during the treatment course [3].

Circulating lymphocytes are characterized by high radiosensitivity, with the dose required to reduce the surviving lymphocyte fraction by 50 % (LD50) as low as 2 Gy [4]. While the negative impact of radiation on lymphocyte counts has been known for over 50 years [5], only recently have we begun to appreciate the crucial role of the immune system in tumour control [6]. Given that cytotoxic CD8+ T lymphocytes are key effectors of immunotherapy, and that irradiation of lymphocyte-rich organs causes transient or sustained lymphopenia, the principle to keep doses ‘As Low As Reasonably Achievable’ was proposed when planning treatment especially in patients receiving immunotherapy [7]. The so-called lymphocyte-sparing RT is currently being investigated in a clinical trial aimed to selectively reduce the irradiation of circulating blood in patients treated for non-small-cell lung cancer (NSCLC) [8]. In a different clinical trial, autologous lymphocyte infusions are tested to mitigate RIL and enhance immune reconstitution [9].

Despite the current lack of data from randomized clinical trials, it seems that recognizing risk factors and adjusting treatment for patients at high risk of radiation-induced lymphopenia (RIL) is crucial for achieving optimal clinical benefit [10]. It seems particularly relevant in light of recent negative results of a clinical trial evaluating immune checkpoint inhibitor durvalumab administered concurrently with chemoradiotherapy and given that RIL was shown to attenuate benefit of durvalumab after CRT for NSCLC [11]. Unfortunately, not enough quantitative evidence is available to establish the relationship between the dose-volume (DV) parameters to critical normal structures and RIL.

Recent studies suggest that the lungs and heart are critical organs affecting the risk and severity of lymphopenia [12]. An example of an approach evaluating multiple parameters at once termed EDRIC (Effective Dose of Radiation to Circulating Immune Cells) has been proposed as a function of mean heart dose, mean lung dose, mean body dose, and number of fractions [13]. Several mathematical models were also proposed to estimate the dose to circulating lymphocytes during RT [14], [15], [16], [17]. Radiotherapy fractionation [18], technique [19], concurrent chemotherapy and treatment modality (protons vs photons [20]) are also important predictors of lymphocyte depletion.

Intensity-modulated radiation therapy (IMRT) and volumetric modulated arc therapy (VMAT) are highly conformal radiotherapy techniques. VMAT is a type of IMRT that was first introduced in 2007 and is characterized by the delivery of radiation from a continuous rotation of the radiation source, allowing to achieve highly conformal dose distributions [21]. Despite some dosimetric benefits [22], [23], VMAT plans are characterized by ‘low dose bath’, where large volumes of healthy tissue receive very low doses of radiation [24]. To date, no clinical studies have directly compared the effects of IMRT and VMAT on radiation doses to structures associated with lymphopenia risk and rates of lymphocyte depletion.

Although many models predicting RIL have been proposed so far [10], most studies used endpoints that were easy to calculate rather than biologically justified and that did not make use of all available information, limiting power and making it difficult to cross-reference the results. The choice to predict absolute lymphocyte count (ALC) one week after treatment [25] or three months after treatment completion [26] is arbitrary and dichotomizing outcome into lymphopenia/no lymphopenia based on CTCAE definition or ‘optimum cutoff’ determined by ROC curve analysis further increases the odds of finding spurious relationships between endpoints and DV/clinical predictors [27], [28]. Lymphocyte nadir (lowest measured ALC count), on the other hand, is not a good outcome for retrospective studies because its value depends strongly on whether the measurement is performed when ALC counts are actually lowest for each patient. Heterogenous timepoint and cutoff definitions across different studies hinder the synthesis of results from individual reports [10].

In this study we analyse differences in treatment plans and cumulative dose-volume histogram (cDVH) parameters for patients undergoing VMAT and IMRT treatments, assessing how these differences might affect RIL severity. We also propose a mechanistically informed endpoint for studies of radiation-induced lymphotoxicity, utilizing all ALC measurements from RT start until a maximum of 45 days for each patient to fit a lymphocyte depletion curve and calculating the area under decay curve and percentage lymphocyte loss relative to baseline, similar to methodology proposed by Ellsworth et al. [29]. We then use this endpoint in addition to lymphocyte nadir to assess the impact of clinical and DV predictors on RIL and evaluate the performance of proposed RIL predictors in patients treated using different RT techniques, with or without concurrent CRT.

## 2 Materials and methods

### 2.1 Patient recruitment and data extraction

This is a retrospective study performed in three radiotherapy centres in Poland (Radiotherapy Department at the Copernicus Memorial Hospital in Łódź, Department of Oncology & Radiotherapy at the Medical University of Gdańsk and the 3rd Radiotherapy and Chemotherapy Department at the Maria Skłodowska-Curie National Research Institute of Oncology, Gliwice branch). The study was approved by Bioethics Committee of the Medical University of Lodz (RNN/102/21/KE). We included patients older than 18 years-old, with histologically or cytologically documented diagnosis of NSCLC treated between 10.2010 and 04.2023, with at least three blood morphology test results, including at least test performed at a maximum of three months before RT start and at least one during RT, and radical-intent radiotherapy (IMRT or VMAT), with or without concurrent CRT. Out of 306 patients with NSCLC included in the final analysis, 181 (59 %) were treated with VMAT and the rest with IMRT; 113 patients (37 %) received concurrent chemoradiotherapy. Median radiation dose in the whole group was 60 Gy (60–66 Gy). The baseline characteristics for patients grouped by treatment modality are summarized in Supplementary Table 1; characteristics are summarized additionally by participating centre in Supplementary Table 2.

Patient and disease-related characteristics including age, gender, tumour histology, TNM staging, the use of concurrent chemotherapy and absolute lymphocyte counts (ALC), neutrophil (ANC) and white blood cell (WBC) counts three months before, during RT and up to 60 days after treatment completion were recorded. ALC count closest to RT start and recorded until second day of treatment was recorded as baseline. Collected radiotherapy regimen information included technique (VMAT vs IMRT), planning tumour volume (PTV), total radiation dose, number of fractions, and cDVH parameters for the heart, sum of lungs and body: minimal dose, maximum dose, mean dose, and the percentage volume of each structure receiving at least x% (Vx) for 0.5, 1, 2 and 5–55 Gy in increments of 5 Gy. The selection of DV predictors was based on previous research [18], [30]. To account for differences in fractionation, each cDVH parameter was converted to equivalent dose of 2 Gy fractions (EQD2) assuming α/β of 10 Gy [31].

EDRIC was calculated according to the formula:

$$\mathrm{EDRIC}=0.12*MLD+0.08*MHD+\left[0.45+0.35*0.85*{\left(\frac{\#of\;fractions}{45}\right)}^{\frac{1}{2}}\right]*MBD$$

where MLD is mean lung dose, MHD is mean heart dose, and MBD is mean body dose.

### 2.2 Statistical analysis

Patient characteristics were described using number with appropriate percentage or median with 25–75 %, unless stated otherwise, and compared between groups using Chi^2^ test, Mann-Whitney *U* test or Kruskal-Wallis test, as appropriate. For treatment plan comparison between VMAT and IMRT, analysis of covariance (ANCOVA) was performed while accounting for PTV volume and the significance of VMAT vs IMRT contrast was extracted.

All included patients had at least three blood morphology test results, including one performed at baseline. To investigate ALC loss dynamics, a curve was fitted separately for each patient from RT onset until a maximum of 60 days, assuming exponential decay function with parameters a and c bounded by 0, since negative ALC levels are non-physiological [29]:

ALC(t)=a∗exp(-b∗t)+c

and the parameter values were recorded for each patient. For the initial parameter values for the fitting procedure, we used values informed by the literature: 0.10 for ALC depletion rate and 0.34 for plateau [32]. Percentage ALC loss was estimated for each patient by calculating the area under the patient-specific exponential decay curve and subtracting it from baseline ALC * last recorded ALC within 45 days from the start of RT (curve fitting time). To compare the relative predictive power of cDVH parameters for lymphocyte loss, we fitted linear regression models based on each parameter separately and concurrent chemotherapy (yes or no) and percentage lymphocyte loss as dependent variable and recorded the model’s R^2^.

Dimensionality reduction was performed for DV predictors principal component analysis (PCA) and the value of first principal component (PC1) was retained. Linear regression models were created for percentage ALC loss based on most important clinical predictors from the literature and univariable analysis (concurrent CRT, baseline ALC) and either EDRIC or PC1. Model performance was described using R^2^, bias-corrected R^2^ (calculated based on 300 bootstrap resamples), mean squared error (MSE) and Akaike Information Criterion (AIC).

Statistical analysis was performed using R version 4.3.1 (packages: DVHmetrics [31], rms [33]) and Python 3. All tests were two-sided and p-values <0.05 were considered significant.

## 3 Results

**Fig. 1:**
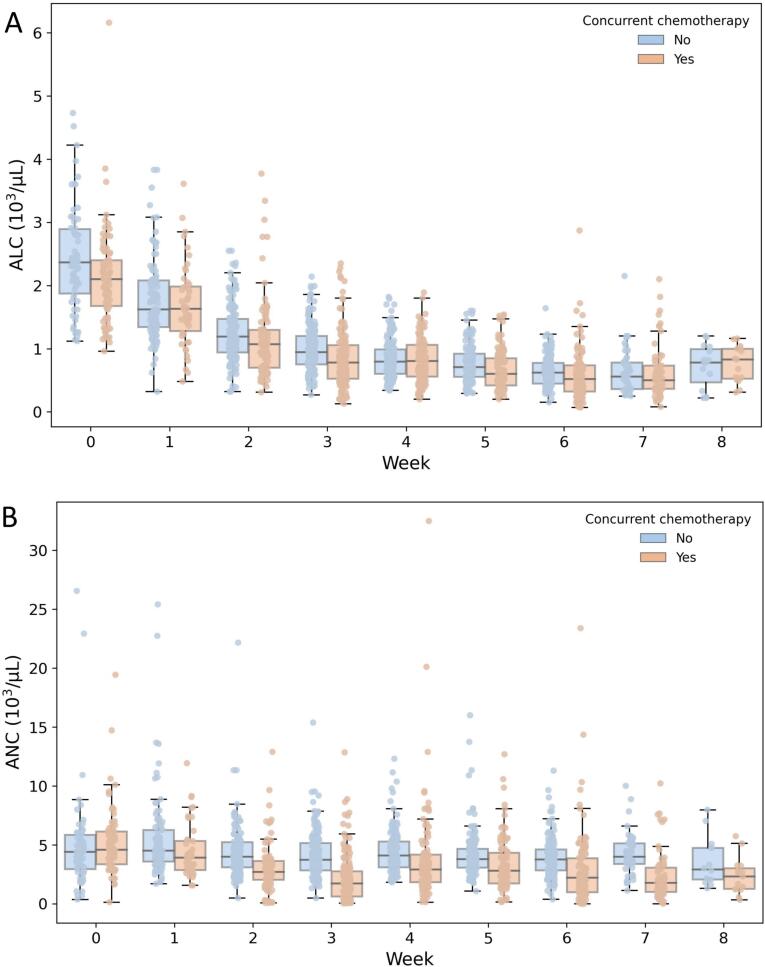
Absolute lymphocyte count (ALC, panel A), and absolute neutrophil count (ANC, panel B), in consecutive weeks since the start of treatment in patients undergoing RT with or without concurrent chemotherapy. Boxes represent medians with 25–75% of data and the whiskers represent 1.5*interquartile range.

Longitudinal dynamics of ALC during treatment course in patients treated with RT with or without concurrent CRT is shown in Fig. 1A. ALC decreased over the treatment course from 2.07*10^9^/L (1.53–2.54*10^3^/µL) at baseline and recovered partially in most patients at week 8 since the treatment start, with 132 (43 %) patients developing grade ≥ 3 lymphopenia according to CTCAE (ALC < 500 cells/mm^3^). The median lymphocyte nadir for the whole group of patients was 0.54*10^3^/µL (0.39–0.75*10^3^/µL). Neutrophil counts remained stable during the treatment course in patients treated with RT alone, compatible with their greater radioresistance, but decreased in patients undergoing CRT and remained at low levels at week 8 since the treatment start (Fig. 1B).

To investigate patient- and treatment-related factors associated with lymphocyte loss during RT while making use of as all the available data, we fitted patient-specific exponential decay curves for ALC counts from the RT start until 45 days. Curve fitting was unsuccessful for 27 patients. Exponential decay parameter b < 0 or > 1 and R^2^ < 0.5 were also considered a fitting failure and were observed in 19 and 10 patients, respectively, resulting in 250 fits available for the analysis. The mean R^2^ of the successful fits was 0.92 (all successful fits shown in Fig. Supplement).

The median percentage ALC loss during the first 45 days since RT start, calculated by subtracting the AUC for each patient from the baseline value multiplied by the fitting time, was 52.2 % (43.7–60.9 %) and was higher in patients undergoing CRT (median 58.9 %) than in patients undergoing RT alone (median 48.6 %; p = 0.001; Fig. S1A). In univariable analysis, patient age, concurrent CRT, RT technique and baseline ALC as well as PTV volume were associated with percentage lymphocyte loss during treatment course (Fig. S1B).

**Fig. 2:**
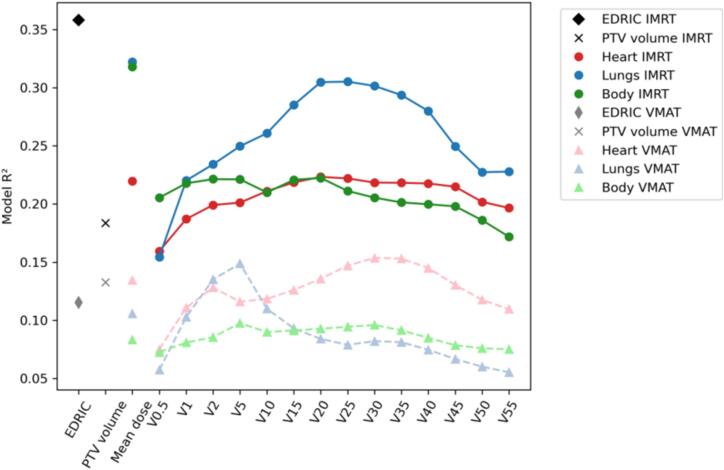
Coefficients of determination (R^2^ scores) for models predicting percentage ALC loss based on concurrent chemotherapy (yes or no) and a single cDVH parameter, categorized by RT technique (IMRT or VMAT). cDVH – cumulative dose-volume histogram, EDRIC – estimated dose of radiation to immune cells, PTV – planning target volume.

In the whole group of patients, the incidence of severe RIL was higher among patients treated with IMRT (51 %) than among those treated with VMAT (37 %; p = 0.012); however, the prescribed radiation dose and median PTV volume were also higher for the IMRT plans and the patients differed in respect to CRT use (Supplementary Table 1). Mean body volume exposed to radiation dose > 2 Gy and > 5 Gy (whole body V2 and V5) was significantly higher for VMAT vs IMRT in ANCOVA adjusted for PTV volume (Fig. S2A), but the difference in volume of lungs exposed to low doses (1–10 Gy) was not significant and IMRT was associated with significantly higher volume of the lungs exposed to higher radiation doses (15 Gy and more) and with bigger heart volume exposure to both low and high radiation doses (1–55 Gy). Mean doses to the sum of lungs, heart and whole body were also higher for IMRT compared with VMAT plans after adjusting for PTV volume. We examined also the correlation structure in cDVH parameters for the heart, sum of lungs, whole body and PTV volume separately for IMRT (Fig. S2B) and VMAT (Fig. S2C); although the correlation structure was similar, correlations between lung-, whole body- and heart-related parameters were slightly stronger for VMAT than IMRT plans (See Fig. 2).

**Fig. 3:**
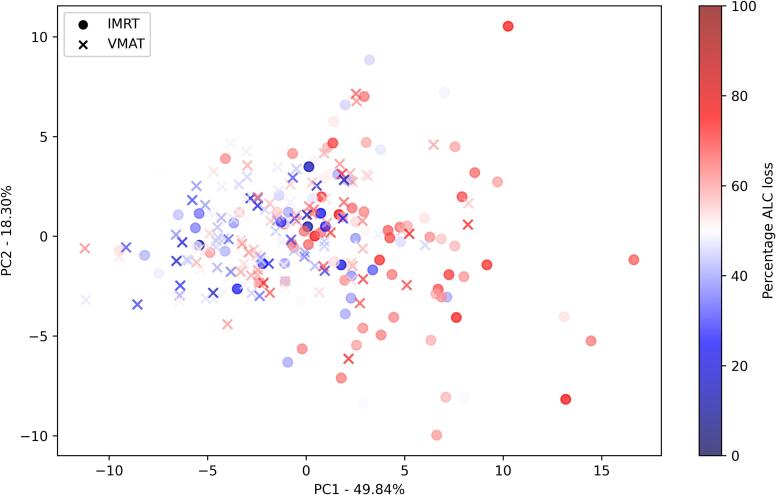
Principal component analysis (PCA) based on dosimetric predictors for the heart, lung sum and whole body colored by percentage ALC loss, with RT technique shown as marker type.

To ensure our method for gauging radiation-induced lymphocyte loss during RT was accurate, we first confirmed that cDVH parameters related more closely to the calculated %ALC loss than to the lymphocyte nadir (absolute Spearman R values ranging 0.23–0.44 vs 0.07–0.32, respectively; Fig.re S3). Then, to compare the relative importance of organs for predicting lymphocyte loss, we built a separate linear regression model for each cDVH parameter with percentage ALC loss as response, controlling also for concurrent chemoradiotherapy as an important predictor of lymphocyte decay (Fig. 3). For IMRT plans, radiation dose to the sum of lungs was more predictive of lymphocyte than doses to the whole body and the heart, with lung volumes irradiated with intermediate doses (V15-V35) being most predictive of lymphocyte loss, along with mean dose to the sum of lungs and whole body. For VMAT plans, heart parameters for intermediate (V25-V35) doses were more important than cDVHs for lungs and whole body. The relationships between DVH parameters (except PTV volume) and %ALC loss were generally linear, with no clear cut-off values visually distinguishing patients with small or large lymphocyte loss (Fig. S4).

To retain the variability in the DVH parameters while accounting for their multicollinearity, we performed dimensionality reduction through principal component analysis (PCA) based on all cDVH parameters for the sum of lungs, heart and body. First principal component (PC1) retained 49.8 % of cDVH data variability (Fig. 3). Both PC1 and EDRIC were significant predictors of %ALC loss (based on the data of 250 patients for whom %ALC loss could be calculated) in univariable analysis (p < 0.001 for both).

In multivariable analysis adjusted for concurrent chemotherapy, PTV volume and baseline ALC, RT technique (VMAT vs IMRT) was not associated with %ALC loss (p = 0.120). A model based on baseline ALC, concurrent chemotherapy use (yes or no) and PC1 (Fig. S5) was more accurate in predicting %ALC loss than a corresponding model based on clinical predictors and EDRIC (Table 1; bias-corrected R^2^ 0.40 vs 0.39).

**Table 1:** Results of linear regression analysis of the association between patient- and treatment-related characteristics with percentage lymphocyte loss during treatment course.

	**Model 1**		**Model 2**	
	**Regression Coefficient** **(95 % CI)**	p	**Regression Coefficient** **(95 % CI)**	p
**Intercept**	16.15 (10.45, 21.85)	**<0.001**	31.36 (27.25, 35.47)	**<0.001**
**Concurrent CRT [yes vs no]**	7.00 (4.16, 9.84)	**<0.001**	7.44 (4.63, 10.24)	**<0.001**
**Baseline ALC [10^3^/µL]**	7.83 (6.05, 9.62)	**<0.001**	7.85 (6.09, 9.60)	**<0.001**
**EDRIC**	2.37 (1.69, 3.05)	**<0.001**	–	–
**DVH PC1**	–	–	1.13 (0.83, 1.43)	**<0.001**

**Model quality comparison**				

	**Model 1**		**Model 2**	

**R^2^**	0.41		0.42	
**Bias-corrected R^2^**	0.39		0.40	
**MSE**	117.2		113.9	
**AIC**	1908		1901	

## 4 Discussion

In this retrospective study we investigated patient- and treatment-related predictors of RIL in patients undergoing RT for NSCLC. We propose a novel measure of RIL, percentage ALC loss during first 45 days of treatment, that makes use of all available lymphocyte counts during the specified period and show that the percentage loss is more highly correlated with DVH parameters for relevant structures than lymphocyte nadir. This method is therefore more robust than single-timepoint assessments, and using a continuous rather than a binary (lymphopenia/no lymphopenia) endpoint decreases the likelihood of finding spurious associations [27], [28]. With this approach, we identify patient- and treatment-related predictors of RIL. We found also that unlike ALC, neutrophil counts remained stable during the treatment course in patients treated with RT alone, compatible with their greater radioresistance [34], but decreased in patients undergoing chemoradiotherapy.

Radiation-induced lymphopenia impairs tumour control and patient outcomes across multiple solid tumours and treatment modalities [35]. In patients with metastatic cancer treated with immune checkpoint inhibitors (ICI), RIL is an independent poor prognostic factor that may attenuate the benefit from ICI [36], [37]. Accurately modelling RIL to fully investigate its’ predictors and improve selection of patients who may not benefit from ICI is therefore of high clinical importance. Correctly identifying the structures that should be considered organs at risk in patients treated with modalities that rely on functionating immune system is also vital to design better treatment approaches that minimize risk of RIL.

An important issue with many of the published approaches to predict RIL is the selection of ‘best’ DVH predictors using stepwise procedures [38] that are known to produce irreproducible results, especially with large number of potential exploratory variables [39]. Previous studies reported that mean lung and heart dose were associated with lymphopenia risk and DVH constraints for these structures were proposed [12]. While we were able to confirm these associations, we found that the relationships between %ALC loss and DVH parameters were generally linear (with exception of PTV volume), with no clear cut-off values distinguishing patients with small vs large lymphocyte loss.

Given the nature of radiation dose deposition within the patient, cumulative DVH (cDVH) values for adjacent dose levels are highly correlated [40]. Multicollinearity does not preclude good model predictive performance for new patients if they are treated with the same or similar treatment technique, where the same type of collinearities is present as in the original data. Here, we show that cDVH parameters better predict RT-induced lymphocyte depletion in patients treated with IMRT and that high RT doses to different structures may be more relevant for VMAT vs IMRT, a result that should be confirmed in an independent patient cohort.

To retain the variability in cDVH parameters between patients while not including multicollinear predictors in the model, we performed PCA on cDVH data for the lung sum, heart and whole body and PTV volume and used the value of the PC1 to predict percentage lymphocyte loss. First principal component was highly predictive of lymphocyte loss in both univariable analysis and after adjusting for concurrent chemotherapy use and baseline ALC, and the multivariate model based on PC1 and these clinical predictors showed good predictive performance. RT technique, in turn, was not independently associated with RIL, in line with previous studies that found no difference in toxicity between patients with lung cancer treated with VMAT vs IMRT [41].

One limitation of our study are between-patient differences in ALC measurement timing, but we attempted do mitigate the associated bias by impact by fitting a lymphocyte depletion curve for each patient. Another limitation is the difference in patient characteristics between participating centers and the fact that we did not take into account the beam-on time, that may also impact the exposure of circulating lymphocytes to radiation [15], or radiation doses to other normal structures. We also did not assess ALC recovery after RT, because of the small number of patients with sufficient post-RT completion counts and potential bias associated with the non-randomness of data availability (since patients in worse general condition are likely to be hospitalized longer and have blood tests performed more often). Although we did not validate the described models in an independent patient group, we used bootstrap to internally validate and compare the bias-corrected model quality.

In conclusion, we propose percentage lymphocyte loss as an endpoint for studies predicting RIL that maximizes the use of available information from ALC counts during RT and statistical power. We then use this endpoint to show that concurrent CRT and DVH parameters are independently associated with lymphocyte loss in patients with NSCLC treated with modern radiotherapy techniques.

## CRediT authorship contribution statement

**Zuzanna Nowicka:** Conceptualization, Data curation, Formal analysis, Investigation, Methodology, Software, Visualization, Writing – original draft. **Kasper Kuna:** Data curation, Formal analysis, Investigation, Software, Validation, Visualization, Writing – original draft. **Mateusz Łaszczych:** Data curation, Investigation, Methodology. **Małgorzata Łazar-Poniatowska:** Resources, Data curation, Investigation. **Bartosz Kamil Sobocki:** Resources, Data curation, Investigation, Writing – review & editing. **Konrad Stawiski:** Methodology, Software, Resources, Data curation. **Michał Dąbrowski:** Data curation, Investigation. **Konrad Bruski:** Data curation, Investigation. **Adam Zięba:** Resources, Investigation. **Mateusz Pajdziński:** Resources, Investigation. **Emilia Staniewska:** Data curation, Investigation, Writing – review & editing. **Marcin Miszczyk:** Data curation, Investigation, Writing – review & editing. **Harald Paganetti:** Methodology, Writing – review & editing. **Wojciech Fendler:** Conceptualization, Funding acquisition, Methodology, Project administration, Resources, Supervision, Validation, Writing – review & editing. **Bartłomiej Tomasik:** Investigation, Methodology, Resources, Software, Supervision, Writing – review & editing.

## Declaration of competing interest

The authors declare that they have no known competing financial interests or personal relationships that could have appeared to influence the work reported in this paper.
