# Supplementary material for: Dose-volume metric-based prediction of radiotherapy-induced lymphocyte loss in patients with non-small-cell lung cancer treated with modern radiotherapy techniques
Source: Phys Imaging Radiat Oncol. 2024 May 27;30:100593. doi: 10.1016/j.phro.2024.100593 (PMC11190719; doi:10.1016/j.phro.2024.100593)

Supplementary Materials

Supplementary Table 1. Patient and treatment characteristics (by RT modality). Nominal variables are shown as numbers with percentage and continuous variables are shown as medians with 25-75%.

| **Variable** | **IMRT (N=125)** | **VMAT (N=181)** | **p** |
| --- | --- | --- | --- |
| **Age [years]** | 65 (60-69) | 68 (62-72) | 0.005 |
| **Sex** |  |  |  |
| **F** | 40 (32%) | 70 (39%) | 0.463 |
| **M** | 85 (68%) | 111 (61%) |  |
| **Histology** |  |  |  |
| **Adenocarcinoma** | 25 (20%) | 61 (34%) | <0.001 |
| **Squamous cell carcinoma** | 52 (42%) | 88 (49%) |  |
| **Adenocarcinoma/ Squamous cell carcinoma** | 1 (1%) | 3 (2%) |  |
| **Large cell neuroendocrine carcinoma** | 1 (1%) | 8 (4%) |  |
| **Large cell carcinoma** | 0 (0%) | 3 (2%) |  |
| **NOS** | 46 (39%) | 18 (10%) |  |
| **T** |  |  |  |
| **0** | 4 (3%) | 5 (3%) | <0.001 |
| **1** | 7 (6%) | 36 (20%) |  |
| **2** | 35 (28%) | 63 (35%) |  |
| **3** | 43 (34%) | 54 (30%) |  |
| **4** | 36 (29%) | 23 (13%) |  |
| **N** |  |  |  |
| **0** | 9 (7%) | 26 (14%) | 0.238 |
| **1** | 21 (17%) | 30 (17%) |  |
| **2** | 82 (66%) | 104 (57%) |  |
| **3** | 13 (10%) | 21 (12%) |  |
| **AJCC stage** |  |  |  |
| **1** | 1 (1%) | 5 (3%) | 0.006 |
| **2** | 6 (5%) | 28 (15%) |  |
| **3** | 118 (94%) | 148 (82%) |  |
| **Concurrent chemotherapy** | 39 (30%) | 74 (40%) | 0.108 |
| **Lymphopenia < 500/µL** | 64 (51%) | 68 (38%) | 0.017 |
| **Prescribed RT dose [Gy]** | 66 (60-66) | 60 (60-66) | <0.001 |
| **N fractions** | 30 (30-33) | 30 (30-33) | <0.001 |
| **Treatment duration [days]** | 43 (41-44) | 42.9 (39-45) | 0.510 |
| **PTV volume [cm^3^]** | 485 (375-639) | 317 (198-436) | <0.001 |
| **Baseline ALC [10^3^/µL]** | 2.1 (1.6-2.6) | 2.0 (1.5-2.5) | 0.509 |
| **Time to baseline ALC [days]** | 6 (1-19.0) | 4.0 (0-36) | 0.475 |
| **Nadir ALC [10^3^/µL]** | 0.5 (0.3-0.7) | 0.6 (0.4-0.8) | 0.008 |

Supplementary Table 2. Patient and treatment characteristics (by centre). Nominal variables are shown as numbers with percentage and continuous variables are shown as medians with 25-75%.

| **Variable** | **Center 1 (Łódź)**  **N=134** | **Center 2 (Gdańsk)**  **N=124** | **Center 3 (Gliwice)**  **N=48** | **p** |
| --- | --- | --- | --- | --- |
| **Age [years]** | 67 (62-71) | 64.88 (60-69) | 65 (62-70) | 0.043 |
| **Sex** |  |  |  |  |
| **F** | 46 (34%) | 42 (34%) | 22 (46%) | 0.298 |
| **M** | 88 (66%) | 82 (66%) | 26 (54%) |  |
| **Histology** |  |  |  |  |
| **Adenocarcinoma** | 53 (40%) | 17 (14%) | 16 (33%) | <0.001 |
| **Squamous cell carcinoma** | 56 (42%) | 59 (48%) | 25 (52%) |  |
| **Adenocarcinoma/ Squamous cell carcinoma** | 4 (3%) | 0 (0%) | 0 (0%) |  |
| **Large cell neuroendocrine carcinoma** | 5 (5%) | 4 (3%) | 0 (0%) |  |
| **Large cell carcinoma** | 3 (2%) | 0 (0%) | 0 (0%) |  |
| **NoS** | 13 (10%) | 44 (35%) | 7 (15%) |  |
| **T** |  |  |  |  |
| **0** | 6 (4%) | 1 (1%) | 2 (4%) | <0.001 |
| **1** | 28 (21%) | 7 (6%) | 8 (17%) |  |
| **2** | 58 (43%) | 24 (19%) | 16 (33%) |  |
| **3** | 35 (26%) | 52 (42%) | 10 (21%) |  |
| **4** | 7 (5%) | 40 (32%) | 12 (25%) |  |
| **N** |  |  |  |  |
| **0** | 17 (13%) | 9 (7%) | 9 (19%) | 0.059 |
| **1** | 22 (16%) | 23 (19%) | 6 (13%) |  |
| **2** | 81 (60%) | 82 (66%) | 23 (48%) |  |
| **3** | 14 (10%) | 10 (8%) | 10 (21%) |  |
| **AJCC stage** |  |  |  |  |
| **1** | 5 (4%) | 0 (0%) | 1 (2%) | <0.001 |
| **2** | 23 (17%) | 1 (1%) | 10 (21%) |  |
| **3** | 106 (79%) | 123 (99%) | 37 (77%) |  |
| **RT modality** |  |  |  |  |
| **VMAT** | 108 (81%) | 35 (28%) | 38 (79%) | <0.001 |
| **IMRT** | 26 (19%) | 89 (72%) | 10 (21%) |  |
| **Concurrent chemotherapy** | 20 (15%) | 61 (49%) | 32 (67%) | <0.001 |
| **Lymphopenia** | 47 (35%) | 64 (52%) | 21 (44%) | 0.026 |
| **Prescribed RT dose [Gy]** | 60 (60-66) | 66 (60-66) | 60 (60-60) | <0.001 |
| **N fractions** | 30 (30-33) | 30 (30-33) | 30 (30-30) | 0.014 |
| **EDRIC [Gy]** | 5.7 (4.5-6.7) | 7.6 (6.-8.9) | 6.0 (4.9-7.1) | <0.001 |
| **Treatment duration [days]** | 43 (41-45) | 42 (39-44) | 43 (41-48) | 0.004 |
| **PTV volume [cm^3^]** | 348 (216-465) | 484 (362-646) | 238 (167-301) | <0.001 |
| **Baseline ALC [10^3^/µL]** | 2.0 (1.5-2.4) | 2.1 (1.6-2.7) | 2.2 (1.7-2.7) | 0.147 |
| **Time to baseline ALC [days]** | 28 (1-50) | 4 (0-12) | 1 (0-2) | <0.001 |
| **Nadir ALC [10^3^/µL]** | 0.6 (0.5-0.8) | 0.5 (0.4-0.7) | 0.6 (0.4-0.8) | 0.005 |
| **Time to nadir ALC [days]** | 7 (5-10) | 9 (6-15) | 15 (7.8-21) | <0.001 |

Supplementary Figure 1. (A) Percentage ALC loss during treatment course vs PTV volume in patients treated with or without concurrent chemotherapy. Box represents median with 25-75% of data and the whiskers represent 5-95% of data. (B) Univariable analysis of parameters associated with percentage ALC loss.


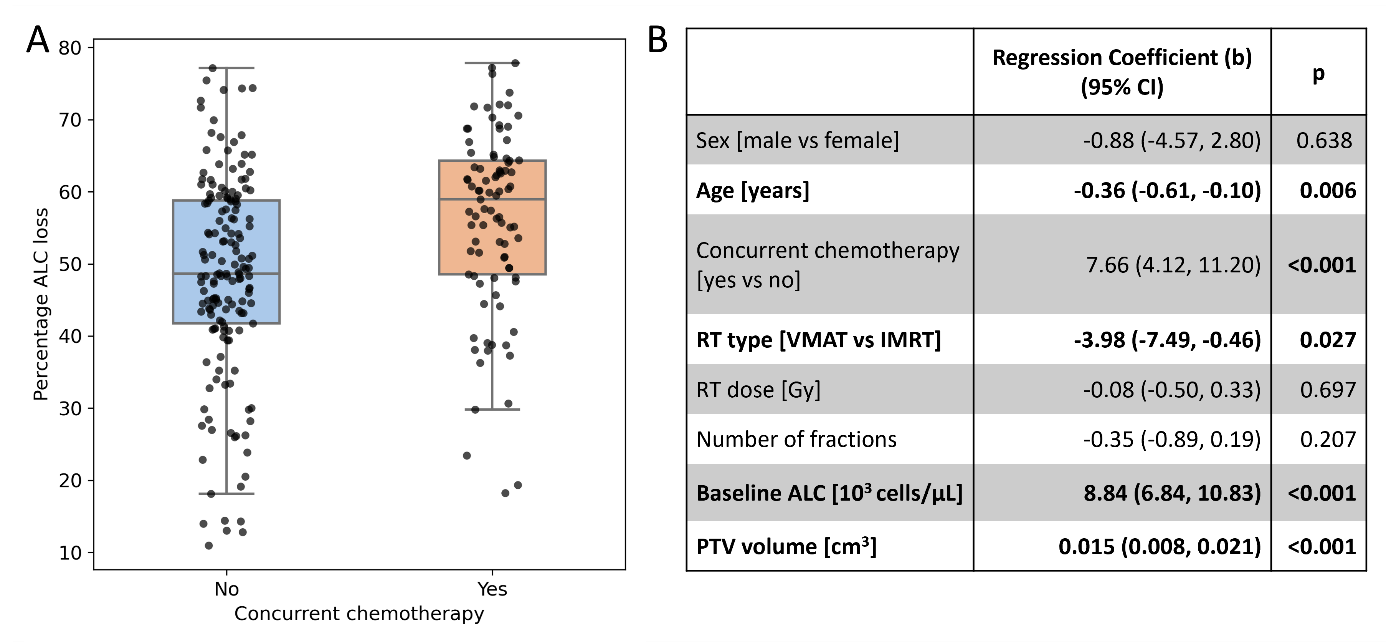


Supplementary Figure 2. Comparison of treatment plans between VMAT and IMRT. (A) Mean values for cDVH parameters for IMRT and VMAT treatment plans; significance is denoted based on analysis of covariance adjusted for PTV volume *** - p<0.001, ** - p<0.01, * - p<0.05. (B) Correlation matrix between cDVH parameters for IMRT and (C) VMAT treatment plans.


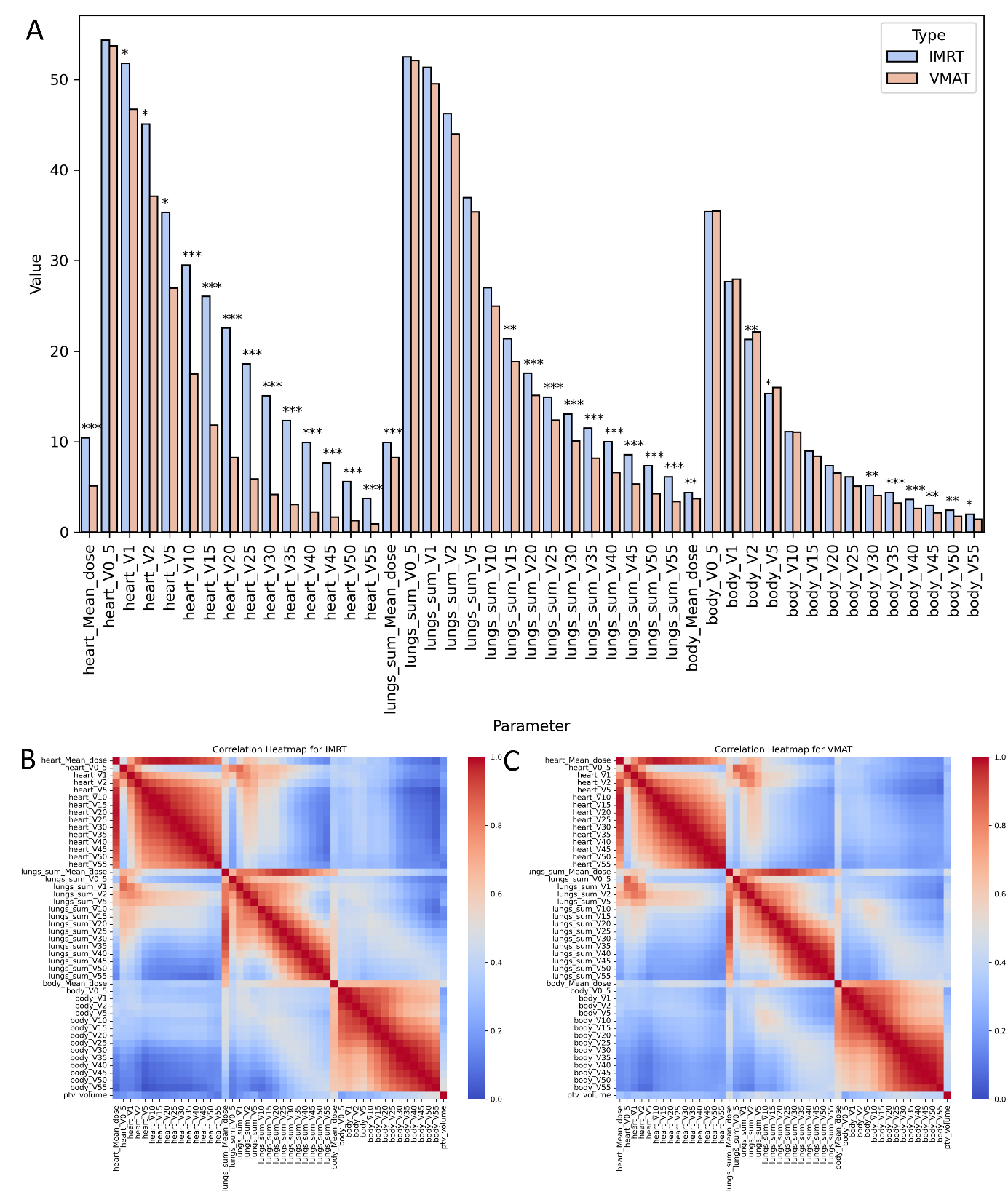


Supplementary Figure 3. Spearman rank correlations between cDVH parameters for the heart, lungs and body and measures of lymphocyte decay: lymphocyte nadir (A) or percentage lymphocyte loss (B).


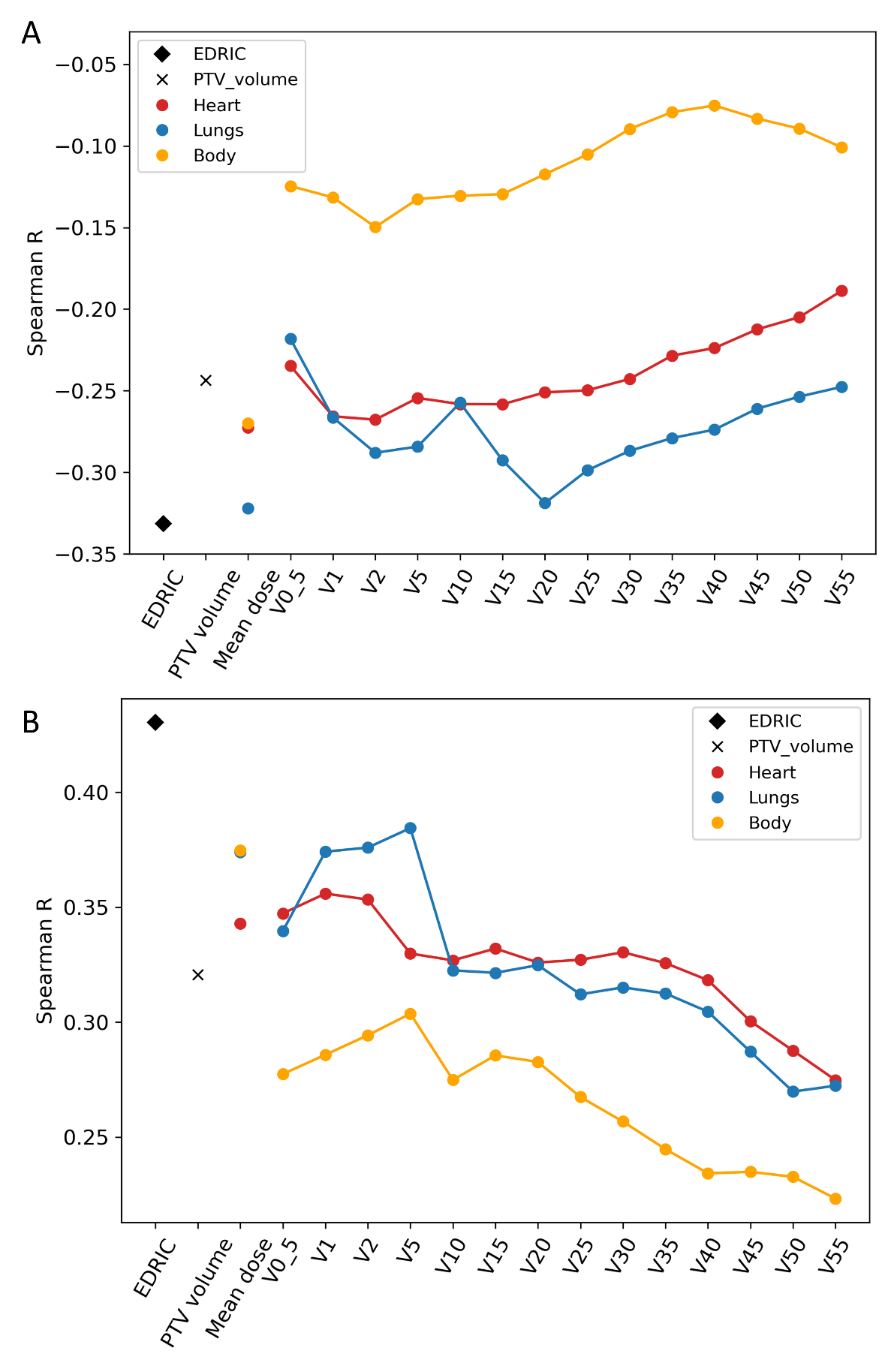


Supplementary Figure 4. Scatterplots of percentage absolute lymphocyte loss vs dosimetric parameters for the heart (red; first row), sum of lungs (blue; second row), body (grey; third row) or planning tumor volume (PTV) volume (black; third row) with locally weighted scatterplot smoothing (LOESS) lines plotted.


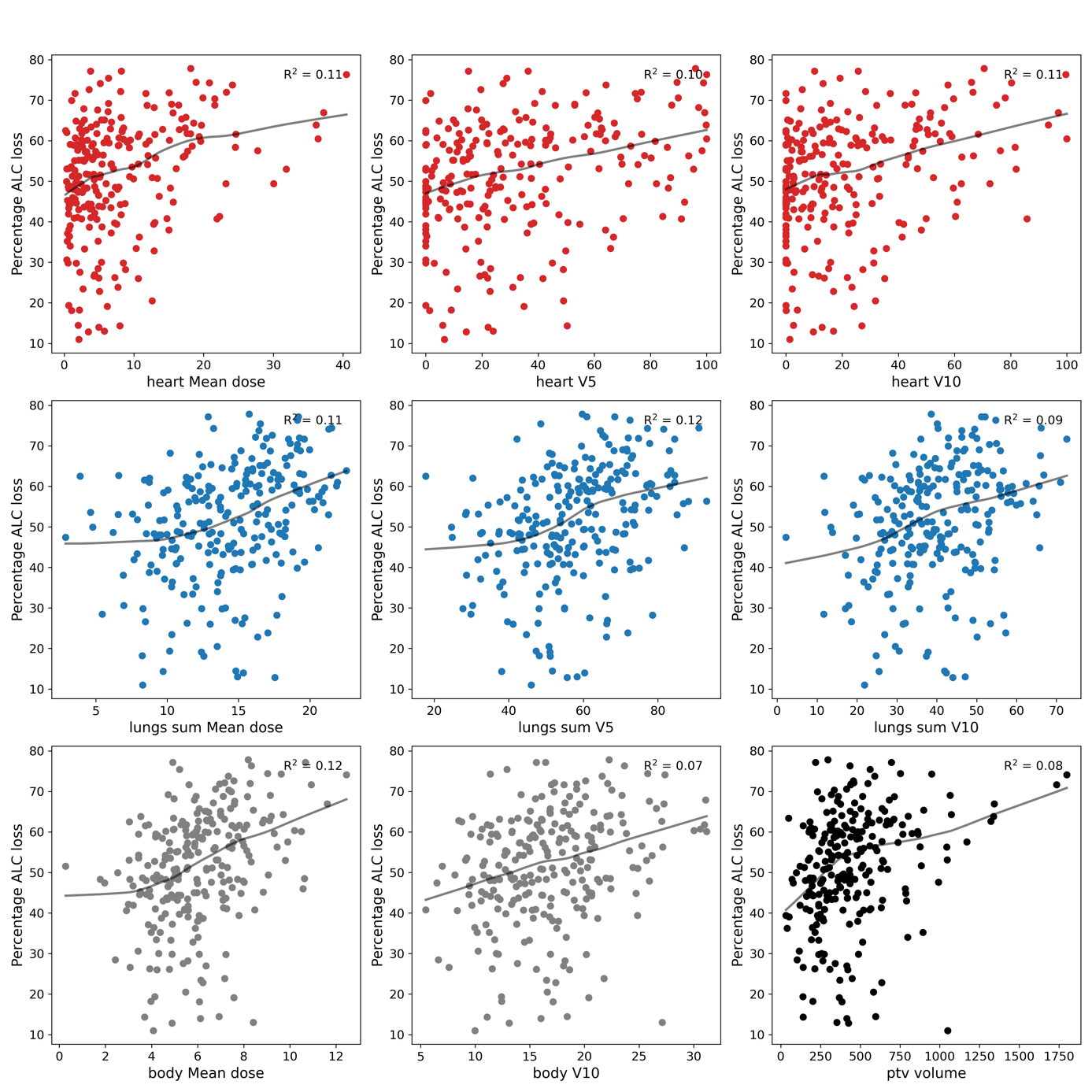


Supplementary Figure 5. Results of model predicting percentage ALC loss based on baseline ALC, concurrent chemoradiotherapy (yes or no) and cDVH parameters. ALC – absolute lymphocyte count


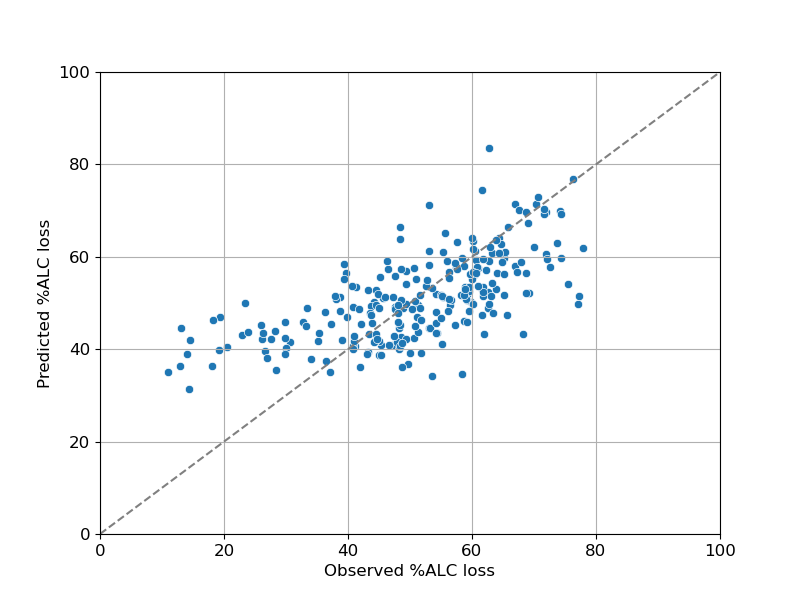


Figure supplement. Results of fitting of exponential decay curve (red) to absolute lymphocyte counts (black dots) of individual patients within 45 days since radiotherapy start.


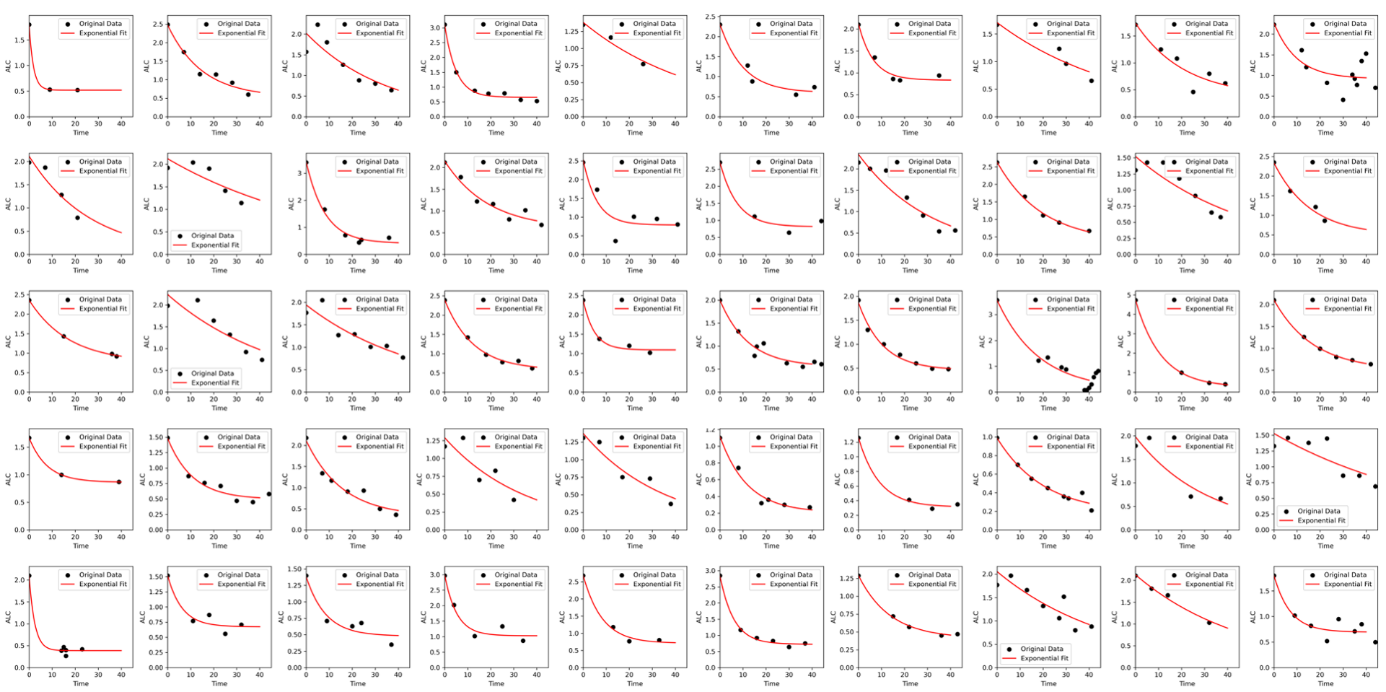

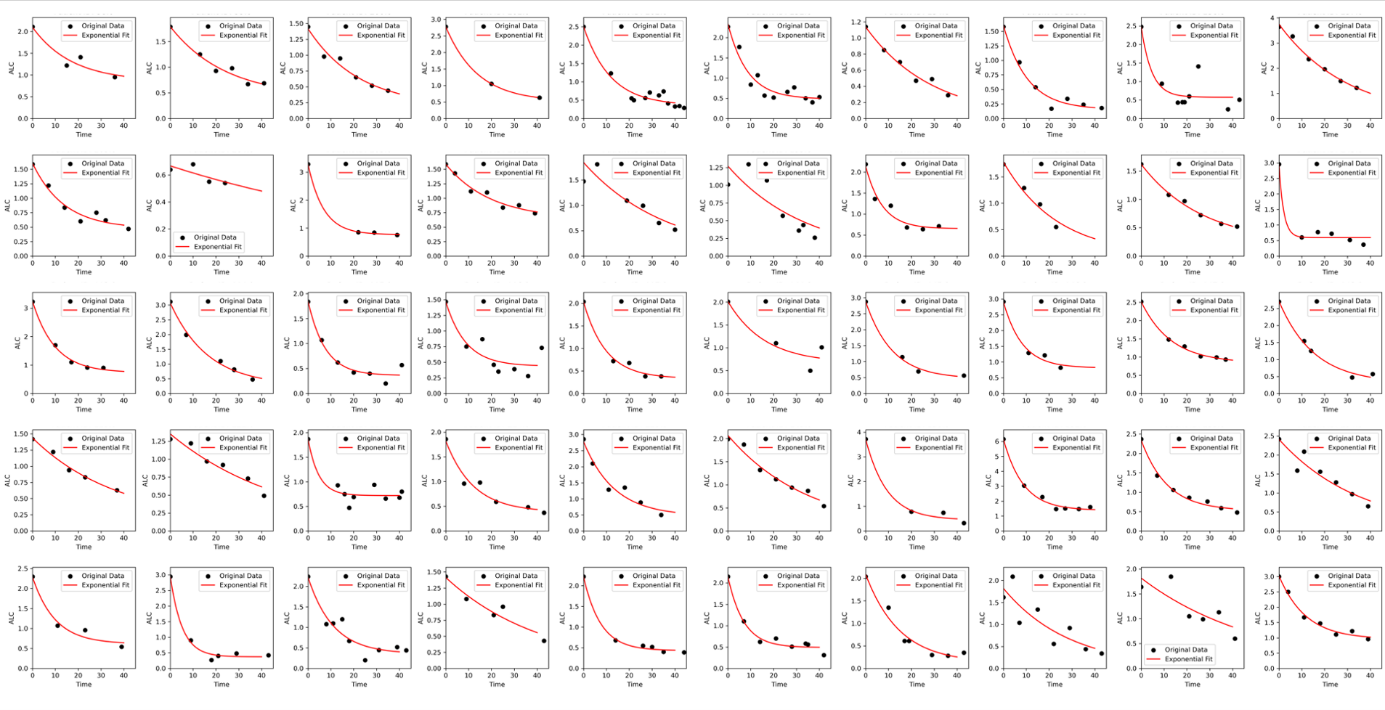

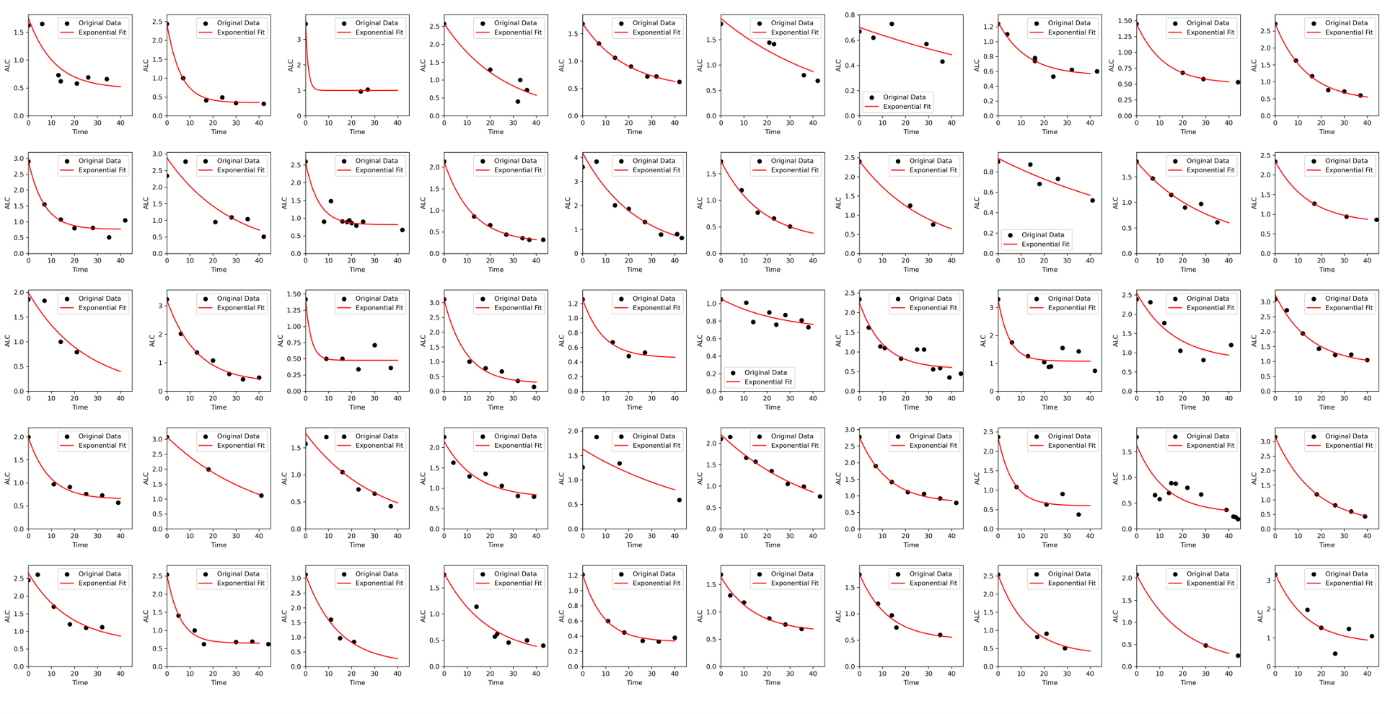

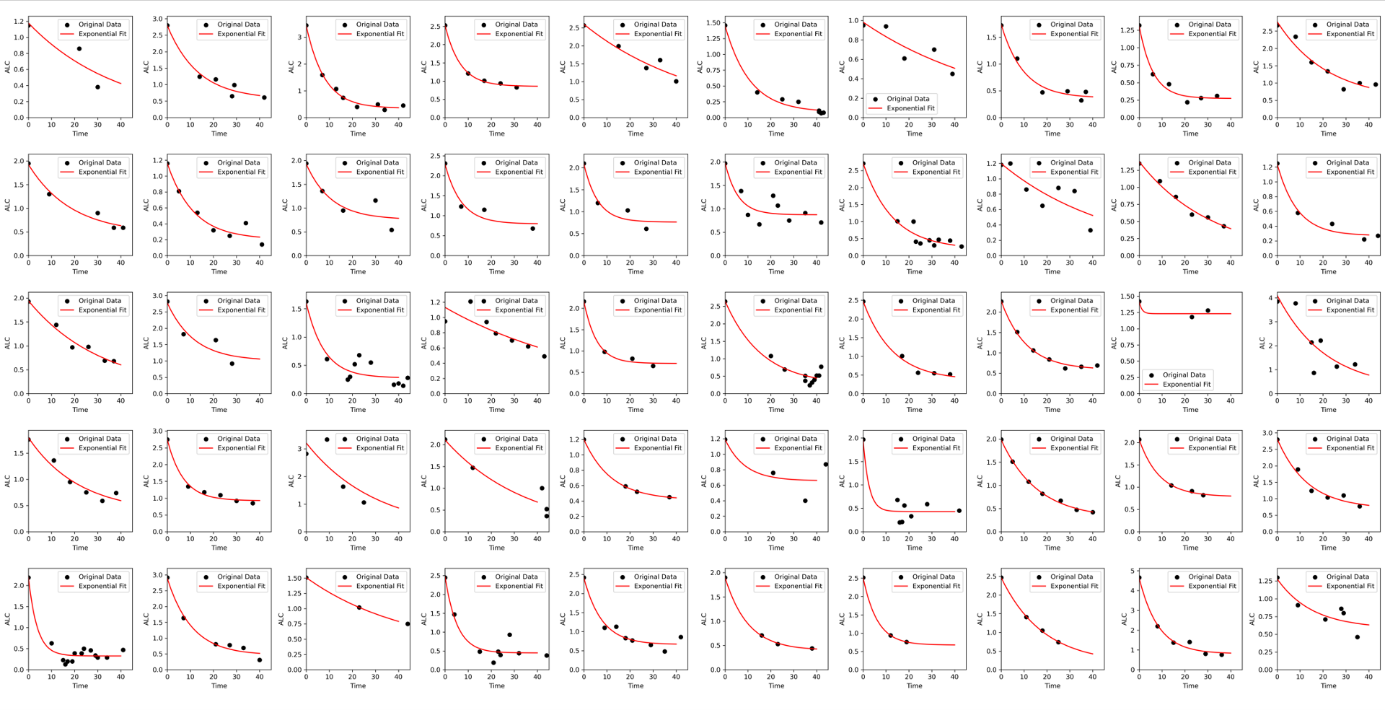


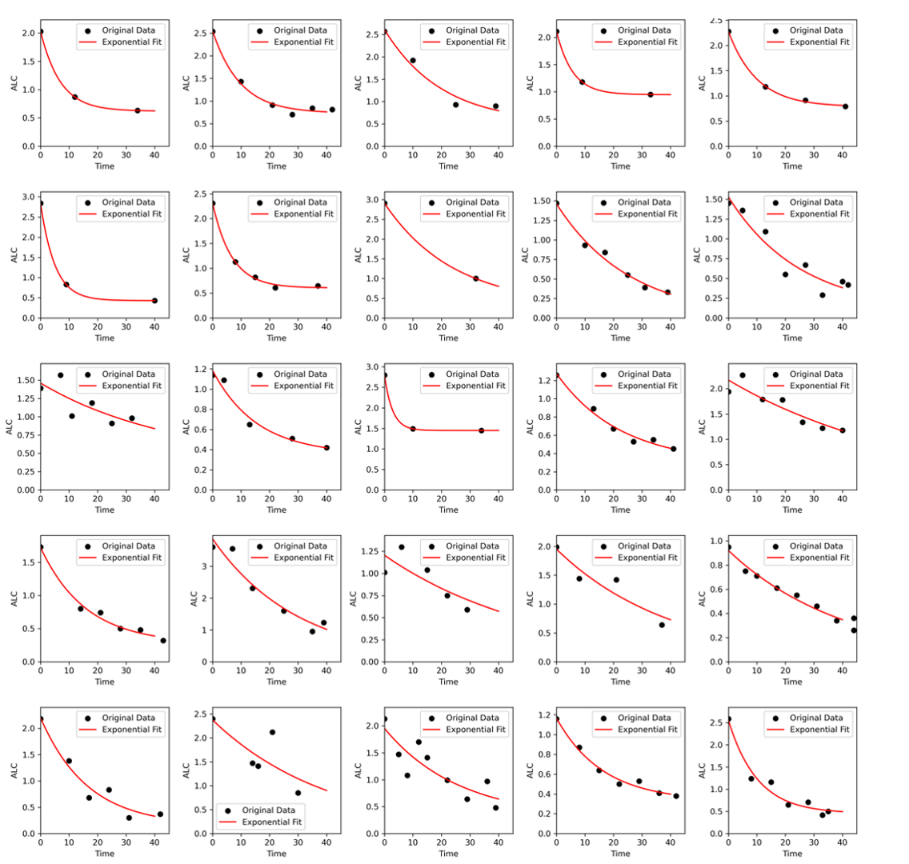

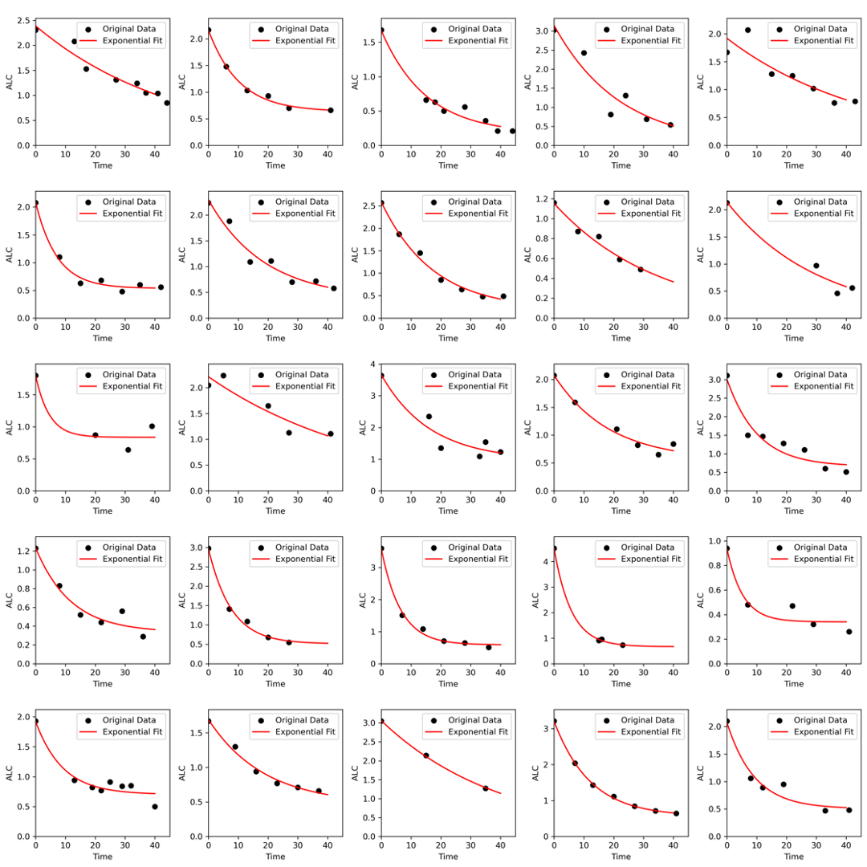

Supplement: Supplementary Data 1 [file mmc1.docx]
